# Blockade of SK4 channels suppresses atrial fibrillation by attenuating atrial fibrosis in canines with prolonged atrial pacing

**DOI:** 10.7150/ijms.69626

**Published:** 2022-11-07

**Authors:** Youcheng Wang, Yajun Yao, Yazhe Ma, Shanqing He, Mei Yang, Zhen Cao, Shiwen Liudi, Yuntao Fu, Huiyu Chen, Xi Wang, Congxin Huang, Qingyan Zhao

**Affiliations:** 1 Xiaogan Hospital Affiliated to Wuhan University of Science and Technology, Xiaogan, China.; 2 Department of Cardiology, Renmin Hospital of Wuhan University Wuhan City, Wuchang, Wuhan, China.; 3 Cardiovascular Research Institute, Wuhan University, Wuhan, China.; 4 Hubei Key Laboratory of Cardiology, Wuhan, China.

**Keywords:** intermediate conductance Ca^2+^-activated K^+^ channel, SK4, TRAM-34, atrial fibrillation, atrial fibrosis, atrial fibroblast

## Abstract

**Background:** We previously found that intermediate conductance Ca^2+^-activated K^+^ channel (SK4) might be an important target in atrial fibrillation (AF).

**Objective:** To investigate the role of SK4 in AF maintenance.

**Methods:** Twenty beagles were randomly assigned to the sham group (n=6), pacing group (n=7), and pacing+TRAM-34 group (n=7). Rapid atrial pacing continued for 7 days in the pacing and TRAM-34 groups. During the pacing, the TRAM-34 group received TRAM-34 intravenous injection (10 mg/Kg) 3 times per day. Atrial fibroblasts isolated from canines were treated with angiotensin II or adenovirus carrying the SK4 gene (Ad-SK4) to overexpress SK4 channels.

**Results:** TRAM-34 treatment significantly suppressed the increased intra-atrial conducting time (CT) and AF duration in canines after rapid atrial pacing (P<0.05). Compared with the sham group, the expression of SK4 in atria was higher in the pacing group, which was associated with an increased number of myofibroblasts and levels of extracellular matrix in atrium (all P<0.05), and this effect was reversed by TRAM-34 treatment (all P<0.05). In atrial fibroblasts, the increased expression of SK4 induced by angiotensin II stimulation or Ad-SK4 transfection contributed to higher levels of P38, ERK1/2 and their downstream factors c-Jun and c-Fos, leading to the increased expression of α-SMA (all P<0.05), and all these increases were markedly reduced by TRAM-34 treatment.

**Conclusion:** SK4 blockade suppressed AF by attenuating cardiac fibroblast activity and atrial fibrosis, which was realized through not only a decrease in fibrogenic factors but also inhibition of fibrotic signaling pathways.

## Introduction

Atrial fibrillation (AF) is known to be one of the most common arrhythmias. In recent years, the incidence and mortality of AF have continued to increase, and AF is mainly associated with ischemic strokes and myocardial fibrotic diseases 1. Although clinically AF can be treated by several ways, such as drugs, catheter ablation and surgical operation, the overall cure rate is not satisfying and the recurrence rate is high in the long term 2,3. Therefore, it is important to clarify the induction and maintenance mechanism of AF to obtain an effective, accurate target to prevent the development of this disease.

The intermediate conductance Ca^2+^-activated K^+^ channel (SK4), genetically coded by *KCNN4*, is a member of the Ca2+-activated K+ channel (KCa) family 4,5. According to the characteristics of their single-channel conductance, KCa family members are classified into large-, intermediate-, and small-conductance K+ channels 6,7. In the last few years, regional and functional distributions of these channels in the heart have been gradually discovered 8,9. It has been demonstrated that the SK4 inhibition significantly reduced delayed afterdepolarizations and calcium transients in the human induced pluripotent stem cell-derived cardiomyocytes obtained from patients with catecholamine-sensitive ventricular tachycardia 10. In our previous study, we found that the expression of SK4 in atria was closely related to AF induction, and that SK4 inhibition significantly suppressed the shortening of atrial effective refractory period (AERP), the increase of dispersion of AERP (dAERP) and the induction of AF in canine after rapid atrial pacing 11. These results suggested that high expression of SK4 in atria might be an important mechanism for triggering cellular activity that results in AF induction.

In terms of AF maintenance, myocardial fibrosis is a pathological process that primarily manifests as an excessive deposition of extracellular matrix (ECM), increased collagen content, and imbalanced synthesis and arrangement of various types of ECM proteins 12. Atrial fibrosis has been considered an important structural change in abnormal atrial conduction and AF persistence, which is beneficial to reentry, transmural conduction and anchoring in drivers of AF 13. Previous studies have demonstrated the close association between SK4 and cardiac fibrosis. Wang et al. 14 found that angiotensin II (Ang II) upregulated SK4 by activating Ang II type 1 receptor (AT1R) and activator protein-1 (AP-1), thus promoting the proliferation of myocardial fibroblasts in rats. Ju et al. 15 also found that SK4 blockade attenuated the ventricular remodeling by inhibiting the proliferation and differentiation of myocardial fibroblasts in mice after myocardial infarction. Recently, She et al. 16 further demonstrated that SK4 expression led to upregulated inflammatory cytokines and promoted the differentiation of bone-marrow derived monocytes into myofibroblasts, leading to myocardial fibrosis in rats. We hypothesized that SK4 channels might play an important role in atrial fibrosis induced by AF, in which the upregulation of SK4 channels could lead to the activation of atrial fibroblasts, thus promoting the progression of atrial fibrosis. In this study, on the one hand, we explored the role of SK4 channels in atrial fibrosis in a canine AF model; on the other hand, we isolated and cultivated fibroblasts from the atria of canines to explore the direct effects of SK4 channels on the activity of atrial fibroblasts.

## Materials and Methods

This study was approved by the animal studies subcommittee of our institutional review board (Laboratory Animal Welfare & Ethics Committee of Renmin Hospital of Wuhan University) and was in compliance with the guidelines of the National Institutes of Health for the care and use of laboratory animals.

### Animal preparation

Twenty beagles with a body weight of 8 to 10 kg were randomly arranged into three groups. The sham group (n=6) received pacemaker implantation under sterile conditions without atrial pacing. The pacing group (n=7) received pacemaker implantation with continuous rapid atrial pacing (450 beats/min) for 7 days before the electrophysiological measurements. The pacing+TRAM-34 group (n=7) underwent the same pacing model as the pacing group while receiving TRAM-34 (MedChemExpress, USA) slow intravenous injection (10 mg/Kg, 3 times per day) for 7 days before the electrophysiological measurements. Detailed methods of cardiac pacemaker implantation were described in our previous work 17. After the operation, all canines were conscious and fed normally. During the procedures of pacemaker implantation and the electrophysiological measurements, each canine was given general anesthesia with sodium pentobarbital (30 mg/kg) and ventilated with room air supplemented with oxygen by a positive pressure ventilator (MAO01746, Harvard Apparatus Holliston, USA), and standard electrocardiogram monitoring and blood pressure were continuously recorded. At the end of the experiment, the animals were euthanized with an intravenous injection of a lethal dose of sodium pentobarbital (150 mg/kg) before recovery.

### Electrophysiological measurements

**Figure 1:**
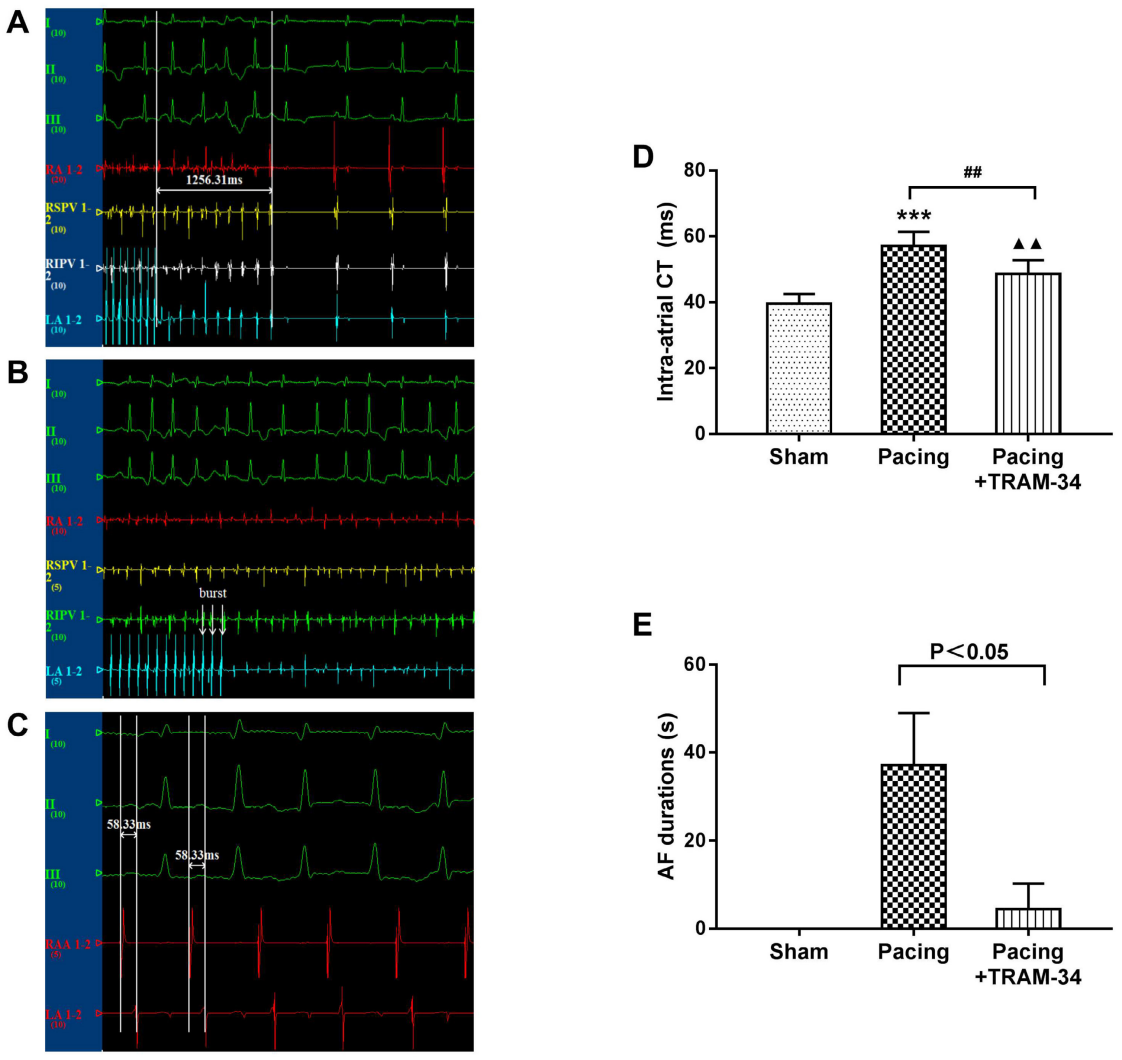
** The results of electrophysiological measurements.** The state that AF was not induced during S1S1 programmed stimulation. (B) The state that AF was induced during S1S1 programmed stimulation. (C) The state that intra-atrial CT was measured. (D) The differences in intra-atrial CT between the groups. (E) The differences in mean AF durations between the groups. ^***^P<0.001 versus the sham group. ^##^P<0.01 versus the pacing group. ^▲▲^P<0.01 versus the sham group. The data are presented as the mean ± SD, n=6, 7, and 7, respectively. **Abbreviations:** CT, conduction time.

All electrophysiological measurements were recorded in a computerized electrophysiology system (Lead 7000, Jinjiang Inc., China). Some original recording processes such as AF induction are shown as **Figure 1A-1C**. Intra-atrial conduction time (CT) was defined as the time difference recorded from the RAA electrode to the low LA electrode during right atrial appendage (RAA) stimulation. An S1S1 programmed stimulus method (120-ms, 100-ms, and 75-ms cycle length, 5 s each, performed 3 times per frequency) was used to evaluate AF durations. AF was defined as an irregular atrial rate faster than 500 bpm lasting for more than 5 s.

### Isolation and culturing of atrial fibroblasts

Atrial fibroblasts were isolated from atria of canines. The atrial tissue were rapidly removed under an aseptic condition and rinsed with cold phosphate buffered saline (PBS), which were then cut into pieces and digested in 0.125 % trypsin for 10 min at 37 °C. Then the pieces were digested using compound enzymes containing 0.25% trypsin and 0.08 % collagenase II for 7-8 times until the tissues were completely digested. After centrifugation (1000 rpm, 5 min), the cells were collected and cultured in an incubator at 37 °C and 5% CO_2_ for 60 min. Cardiomyocytes and fibroblasts were isolated by differential adhesion method. After adhesion of fibroblasts to the bottom of the culture disk, the non-adherent cardiomyocytes were removed from cell plates. The isolated and purified fibroblasts were further incubated in DMEM/F12 at 37 °C in a humidified atmosphere with 5% CO_2_. The passage culture was made at 80 % confluence. The third generation of fibroblasts were inoculated into pore plates for subsequent research. Immunofluorescence of vimentin and fibroblast specific protein 1 (FSP1) was used for fibroblast identification and purity analysis.

### Cells treatment

When cells reached 70-80 % confluence, atrial fibroblasts were cultured with the medium containing 1 uM Ang II with or without 10 uM TRAM-34 for 48 h. To overexpress SK4, cells were infected with the adenovirus (GeneChem, China) carrying the SK4 gene (Ad-SK4) or a control vector (Ad-GFP) at a multiplicity of infection of 100 for 4 h. The medium was replaced by fresh medium with or without 10 uM TRAM-34 after 4 h. After 48 h, the atrial fibroblasts were harvested for further experiments. Optical and fluorescence microscopy (BX51 Systems, Olympus Corporation, Japan) was performed to observe the cells. Ad-GFP and Ad-SK4 were measured as 2x10^11^ plaque-forming units/ml.

### Patch clamp technique

All current recording experiments were conducted at room celsius (20-23 ℃). Cultured atrial fibroblasts for canines were isolated and grouped as described by previous steps. For TRAM-sensitive current recording, pipette solution contained (in mM): K-aspartate 120, KCl 20, MgCl_2_ 1, EGTA 5, Na-phosphocreatine 5, Mg-ATP 5, HEPES 10, free Ca^2+^ 0.8 μM, PH 7.2 adjusted with KOH. The external solution contained (in mM): NaCl 136, KCl 5.4, MgCl_2_ 1, CaCl_2_ 1.8, HEPES 10, Glucose 10, PH 7.4 adjusted with NaOH. The cleaned and polished borosilicate glass electrodes (1.2 mm outer diameter, 100 mm long) were pulled with an electrode pulling device (SUTTER 2000, USA) and had a resistance of 4-7 MΩ when filled with pipette solution. The cells were placed in the cell chamber on an inverted microscope and then filled with extracellular fluid. Under the microscope, the microelectrode tip was sealed with high resistance to the cell membrane by negative pressure attraction. After reaching the gigaohm sealing, the whole-cell configuration was established by gently rupturing the cell membrane under negative pressure. TRAM-sensitive current was initially elicited at a holding potential of -70 mV and then recorded with voltage ramps from -100 mV to +100 mV (10 mV increments per 300 ms). Signals were digitized at 5 kHz and filtered at 2 kHz. Currents were recorded by Pulse software, and the data were analyzed by Clampfit software.

### Histology and immunofluorescence

After measurements were taken, the heart was quickly removed and washed with PBS. The atrial tissue was isolated and fixed with 4% paraformaldehyde, embedded in paraffin and sectioned into 5 microns slices. The cultured fibroblasts were fixed with 4% paraformaldehyde for 15 min, permeabilized with 0.2% Triton X-100 for 20 min, and finally blocked with 3% BSA for 60 min at room temperature.

Atrial fibrosis was quantified by analysis of images of Masson's trichrome-stained samples. The atrial samples were incubated with primary antibodies against SK4 (Abcam, USA), α-SMA (Proteintech Group Inc., China), and DDR-2 (Santa Cruz Biotechnology, USA) and subsequently with secondary antibodies FITC-conjugated AffiniPure goat anti-rat IgG and CY3-conjugated AffiniPure goat anti-rabbit IgG (ASPEN Biotechnology, China). Fibroblasts were incubated with primary antibodies against SK4 and α-SMA and subsequently with secondary antibodies CY3-conjugated AffiniPure goat anti-rabbit IgG and FITC-conjugated AffiniPure goat anti-rat IgG (ASPEN Biotechnology, China). The nucleus was stained with DAPI. The results were analyzed by Image-Pro Plus 6.0 software (Media Cybernetics, USA). Three visual fields in each sample were assessed randomly at 400× magnification.

### Enzyme-linked immunosorbent assay (ELISA)

Blood samples were obtained from the jugular vein separately at baseline and 7 days after atrial pacing. Tissue samples from right atria were obtained after measurements were taken. All the samples were temporarily preserved at -80 °C until testing. Then, samples from all groups were assigned to measure the levels of norepinephrine (NE) and Ang II examined by the corresponding ELISA kit (all from ELK Biotechnology, China) based on the protocol provided by the manufacturer.

### Western blotting

The expression of SK4 channels, ECM proteins, fibrotic factors and the transcription factors in right atria and atrial fibriblasts were measured by Western blotting. The membranes containing the sample proteins were incubated overnight with the primary antibodies against SK4 (Bioss, USA), collagen I, collagen III, matrix metalloproteinase (MMP)-2, MMP-9 (Abcam, USA), transforming growth factor β1 (TGF-β1) (Proteintech Group Inc., China), connective tissue growth factor (CTGF) (Proteintech Group Inc., China), Smad2 and phospho-specific Smad2 (p-Smad2), Smad3 and phospho-specific Smad3 (p-Smad3) , p38 and phospho-specific p38 (p-p38), ERK1/2 and phospho-specific ERK1/2 (p-ERK1/2) (Cell Signaling Technology, USA), c-Fos (Abcam, USA) and c-Jun (Abcam, USA) at 4 °C. Then, they were washed in TBST three times (for 5 min each time), incubated with the diluted secondary antibody for 1 h at room temperature, and imaged using Immun-Star horseradish peroxidase substrate. To ensure that the loaded samples were of equal concentration, the ratio of band intensity to GAPDH was obtained to quantify the relative expression of these proteins.

### Statistical analysis

All data obeyed normal distribution characteristics and are shown as the mean ± standard deviation. Two-sample independent Student's t-test was used to compare the means of two groups. ANOVA followed by Newman-Keuls test was used to compare the mean values of continuous variables among multiple groups, and any significant differences were further analyzed by Tukey-Kramer test. All the statistical tests were two-sided, and a probability value <0.05 was the criterion for statistical significance. All of the statistical analyses were performed with SPSS 20.0 software.

## Results

### Blockade of SK4 channels shortened the intra-atrial CT and AF durations after the prolonged rapid atrial pacing

As shown in **Figure 1,** the intra-atrial CT was significantly increased in the pacing group compared with the sham group (P<0.001) and was dramatically reduced by TRAM-34 treatment (P<0.01). In addition, TRAM-34 treatment significantly reduced the AF durations in canines after prolonged rapid atrial pacing (P<0.05). During the measurement, one canine in the pacing + TRAM-34 group was induced to persistent atrial tachycardia instead of AF, which was rapidly reversed by additional injection of TRAM-34.

### The expression of SK4 channels increased after prolonged rapid atrial pacing

**Figure 2:**
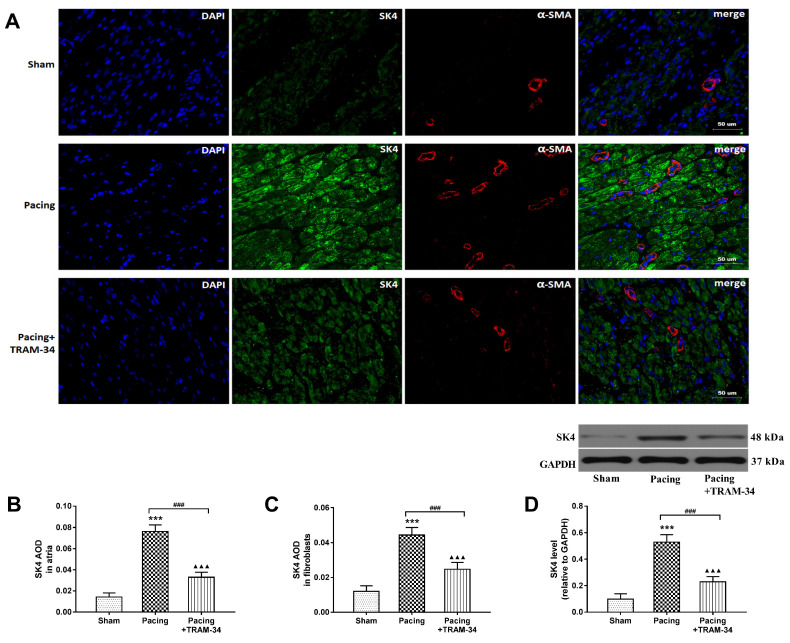
** The expression of SK4 channels in atria.** (A) Double-labeling immunofluorescence staining of SK4 (green) and α-SMA (red) in right atria (400×). (B) SK4 AOD in atria (C) SK4 AOD in cardiac fibroblasts. AOD= IOD/area. (D) Representative Western blot images and the mean levels of SK4 channels in right atria. ^***^P<0.001 versus the sham group. ^###^P<0.001 versus the pacing group. ^▲▲▲^P<0.001 versus the sham group. The data are presented as the mean ± SD, n=6. **Abbreviations:** AOD, average optical density; IOD, integral optical density.

Western blot analysis and double-labeling immunofluorescence staining of SK4 and α-SMA were performed to evaluate the expression of SK4 in right atria. Western blotting showed that the level of SK4 in atria was significantly increased in the pacing group compared with the sham group and the pacing + TRAM-34 group (both P<0.001) (**Figure 2D**). Furthermore, immunofluorescence showed that the average optical density (AOD) of SK4 in atria and fibroblasts was significantly higher in the pacing group than in the sham group (both P<0.001), which could be reversed by TRAM-34 treatment (both P<0.001) (**Figure 2A-2C**). The results indicated that SK4 was commonly expressed in atria and significantly upregulated by rapid atrial pacing.

### Blockade of SK4 channels suppressed myofibroblast proliferation and atrial fibrosis after prolonged rapid atrial pacing

**Figure 3:**
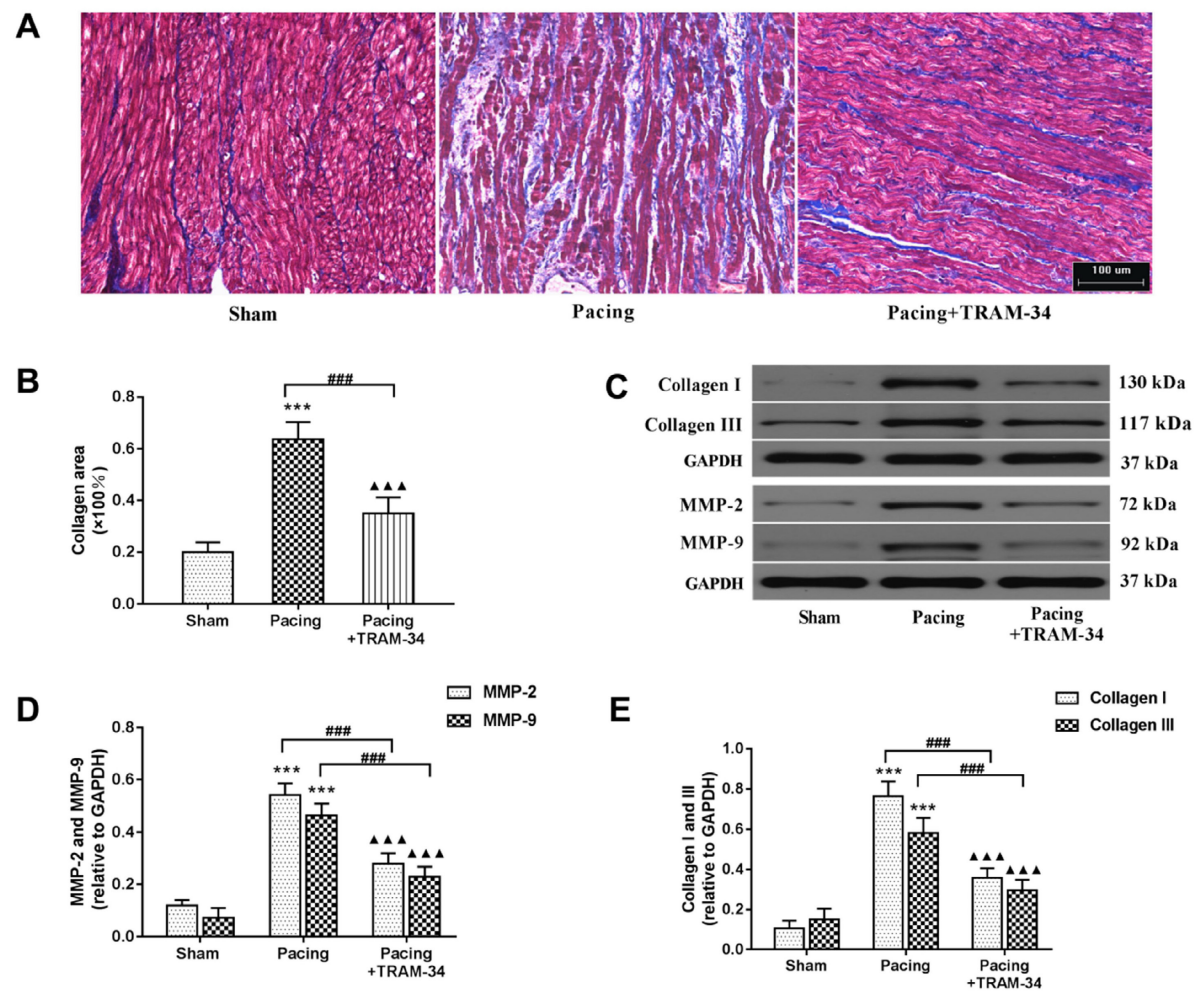
** The expression of collagen and MMPs in atria.** (A and B) Masson's trichrome staining of the atria and mean values of collagen area in the atria (200×). (C-E) Representative Western blotting images and mean levels of collagen I, collagen III, MMP-2 and MMP-9 in the atria. ^***^P<0.001 versus the sham group. ^###^P<0.001 versus the pacing group. ^▲▲▲^P<0.001 versus the sham group. The data are presented as the mean ± SD, n=6.

**Figure 4:**
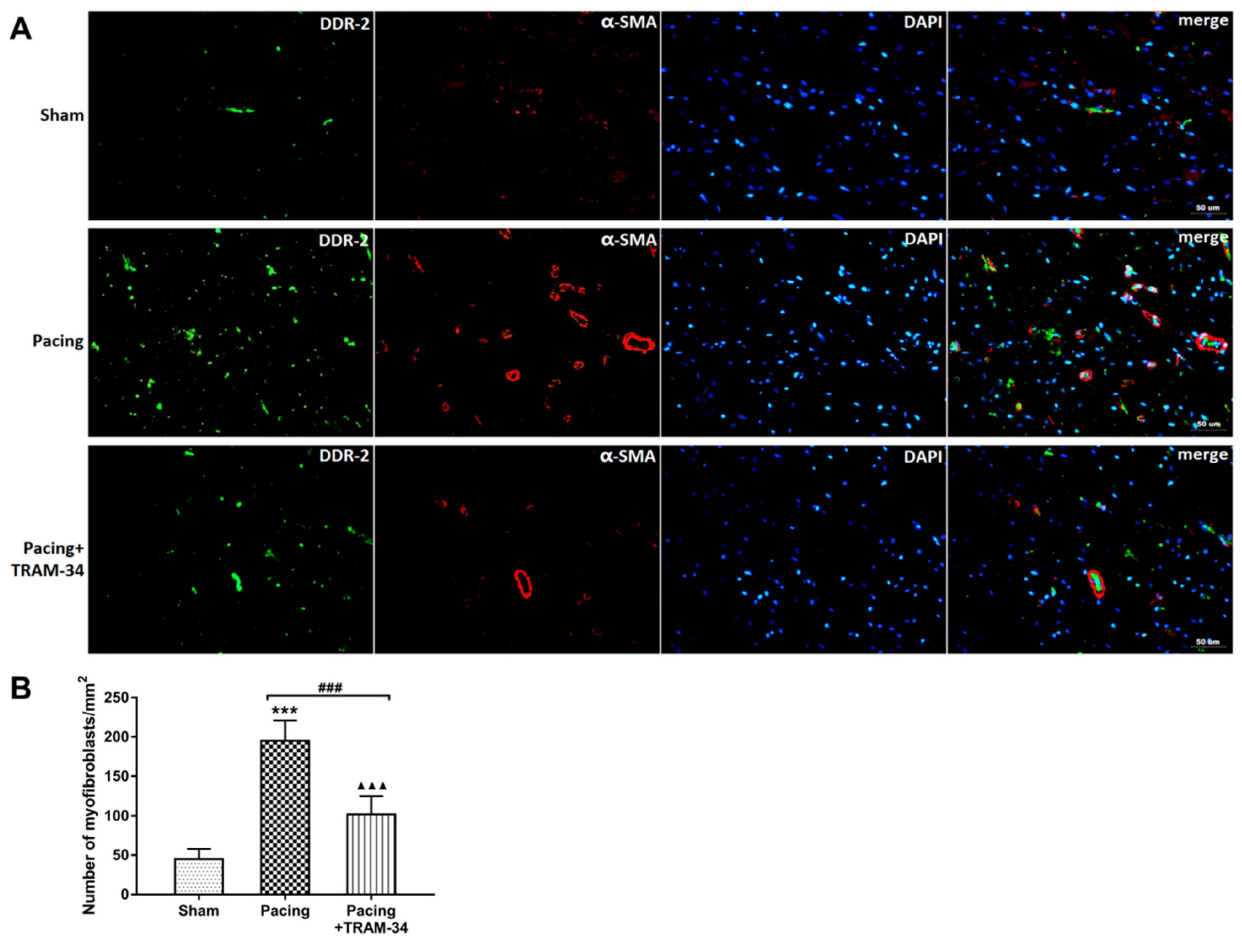
** Colocalization of DDR-2 and α-SMA in atria.** (A) Immunofluorescence staining of myofibroblast markers DDR-2 (green) and α-SMA (red) in the atria (400×). (B) The numbers of myofibroblasts in the atria. ^***^P<0.001 versus the sham group. ^###^P<0.001 versus the pacing group. ^▲▲▲^P<0.001 versus the sham group. The data are presented as the mean ± SD, n=6.

Masson's trichrome staining and Western blot analysis were performed to evaluate the degree of atrial fibrosis. As shown in **Figure 3A** and **3B**, Masson's trichrome staining of atria indicated that the collagen content in these atria was significantly increased in the pacing group compared with the sham group (P<0.001) and was dramatically reduced by the administration of TRAM-34 (P<0.001). Western blotting of collagen I, collagen III, MMP-2 and MMP-9 confirmed this result. Compared with the sham group, the levels of collagen I, collagen III, MMP-2 and MMP-9 in the atrium were much higher in the pacing group (all P<0.001) and were significantly reduced in the pacing + TRAM-34 group (all P<0.001) (**Figure 3C**, **3D** and **3E**). To further explore the effects of SK4 on cardiac fibroblast activation after rapid atrial pacing, immunofluorescent double-labeling staining of the myofibroblast markers α-SMA and DDR-2 in atria was measured. **Figure 4** shows that the number of double-positive cells, with both markers stained, was greatly increased in the pacing group (P<0.001) and was dramatically reduced by TRAM-34 treatment (P<0.001). These results indicated that the SK4 upregulation caused by rapid atrial pacing played a role in promoting atrial myofibroblast proliferation and thereby contributed to the excessive deposition of ECM in atria.

### Upregulated expression of SK4 channels contributed to the activation of atrial fibroblast

**Figure 5:**
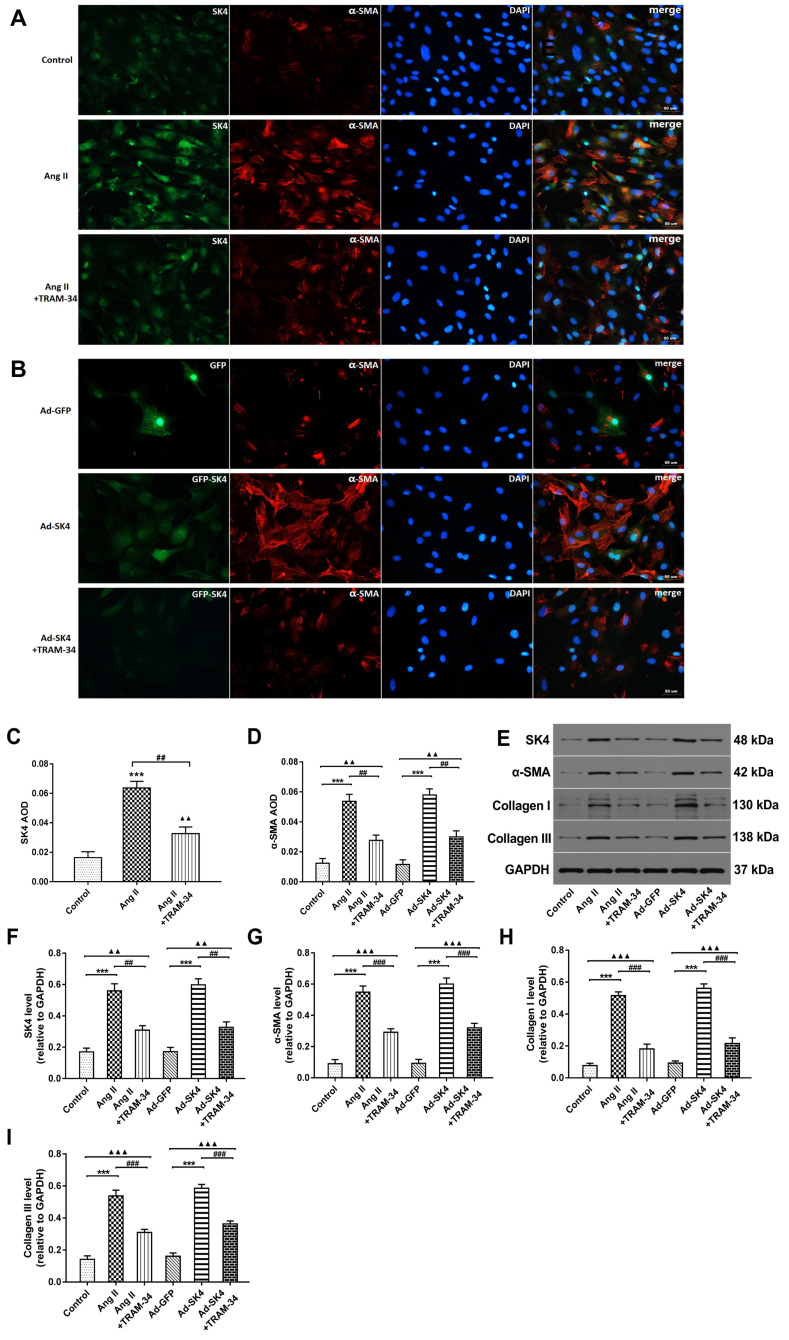
** The role of SK4 channels in the activity of atrial fibroblasts from canines.** (A) Immunofluorescence staining of SK4 (green) and α-SMA (red) in atrial fibroblasts in the Ang II-treated groups (400×). (B) Immunofluorescence staining of α-SMA (red) in atrial fibroblasts in the Ad-GFP (green)-treated groups (400×). (C) The values of SK4 AOD in the Ang II-treated groups. ^***^P<0.001 versus the control group. ^##^P<0.01 versus the Ang II group. ^▲▲^P<0.01 versus the control group. (D) The values of α-SMA AOD in all the groups of atrial fibroblasts. AOD= IOD/area. (E-I) Representative Western blot images and mean levels of SK4, α-SMA, collagen I and collagen III in all the groups of atrial fibroblasts. ^***^P<0.001; ^##^P<0.01; ^###^P<0.001; ^▲▲^P<0.01; ^▲▲▲^P<0.001. The data are presented as the mean ± SD, n=6.

*In vitro*, atrial fibroblasts were treated by Ang II simulation or Ad-SK4 transfection to overexpress SK4. We subsequently used immunofluorescence staining and Western blot to evaluate the SK4 express and used patch clamp technique to exam the TRAM-sensitive current density. The immunofluorescence staining showed that the SK4 density was significantly increased in the Ang II group than in the control group (P<0.001) and was dramatically reduced by TRAM-34 treatment (P<0.01) (**Figure 5A** and **5C**). In the Ad-SK4 group, the fluorescence intensity of GFP coupled with SK4 was significantly higher than that in the TRAM-34 group (**Figure 5B**). Western blotting of SK4 supported the results (**Figure 5E** and **5F**).

**Figure 6:**
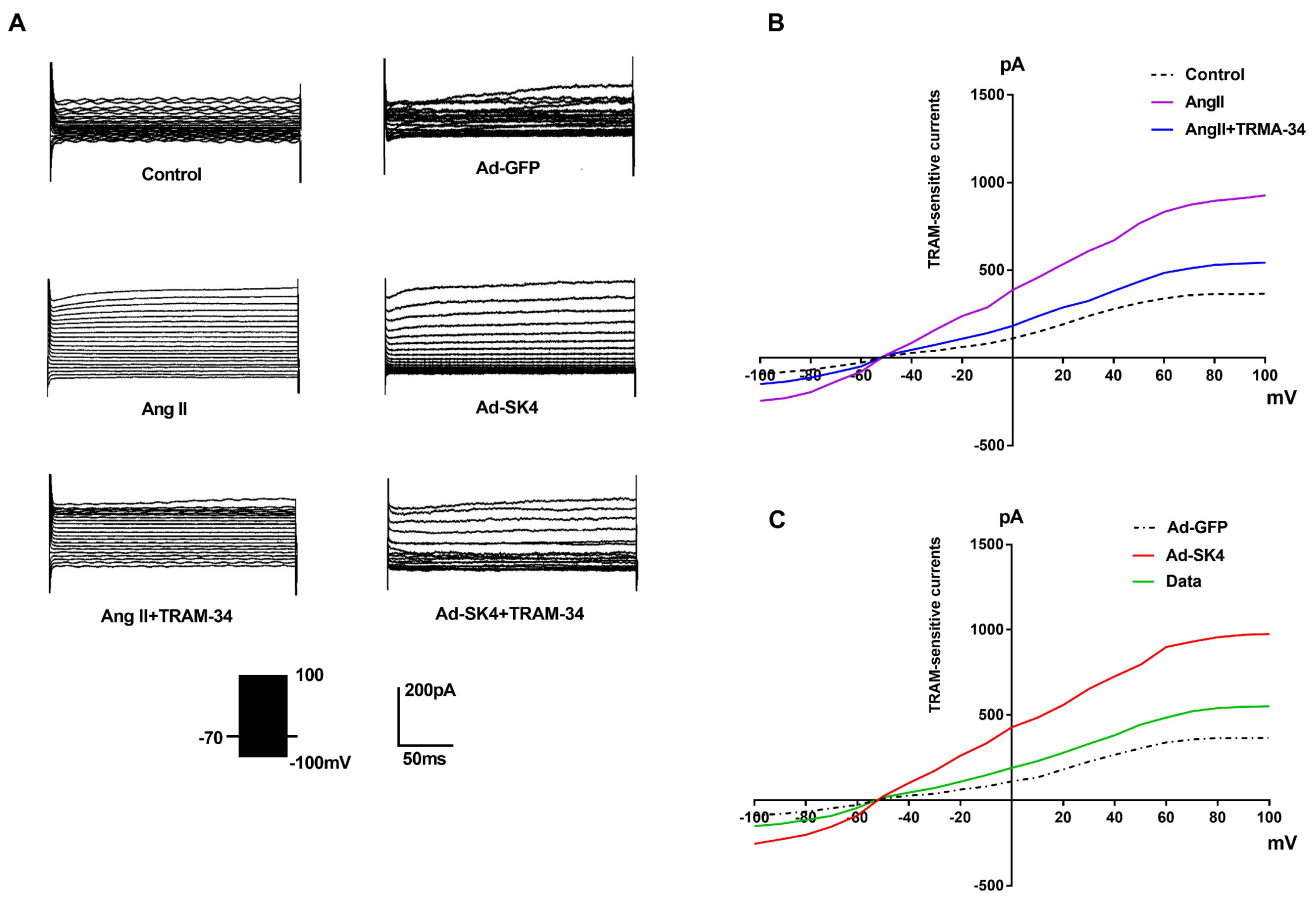
** TRAM-sensitive currents in atrial fibroblasts from canines detected by patch clamp.** (A) Membrane currents recorded in isolated atrial fibroblasts from canines in different groups. (B) Current-voltage relationships of the mean values of TRAM-sensitive currents (pA) in the control group, the Ang II group and the Ang II + TRAM-34 group. (C) Current-voltage relationships of the mean values of TRAM-sensitive currents (pA) in the Ad-GFP group, the Ad-SK4 group and the Ad-SK4 + TRAM-34 group.

As shown in **Figure 6**, the step voltage of patch clamp ranged from -100 mV to +100 mV. The analysis showed that the maximum outward current in Ang II group was significantly higher than that in the control group (P<0.001). Meanwhile, the maximum outward current in the Ad-SK4 group was significantly higher than that in the Ad-GFP group (P<0.001). However, the administration of TRAM-34 significantly reduced the maximum outward current in the Ang II and the Ad-SK4 groups (P<0.001).

To evaluate cardiac fibroblast activation, we examined the myofibroblast marker α-SMA by immunofluorescence staining and Western blotting. Immunofluorescence staining showed that the density of α-SMA was significantly increased in the Ang II and Ad-SK4 groups compared with the control and Ad-GFP groups (P<0.001) and was dramatically reduced by TRAM-34 treatment (P<0.01 ) (**Figure 5A**, **5B** and **5D**). Western blotting also supported these results (**Figure 5E** and **5G**). The levels of collagen I and III were examined by Western blotting to further explore the effects of SK4 on cardiac fibroblast activation. The levels of collagen I and III were much higher in the Ang II and Ad-SK4 groups (both P<0.001) and were significantly reduced by the administration of TRAM-34 (both P<0.001) (**Figure 5E**, **5H** and** 5I**).

### Blockade of SK4 channels reduced the levels of NE, Ang II, CTGF and TGF-β1

**Figure 7:**
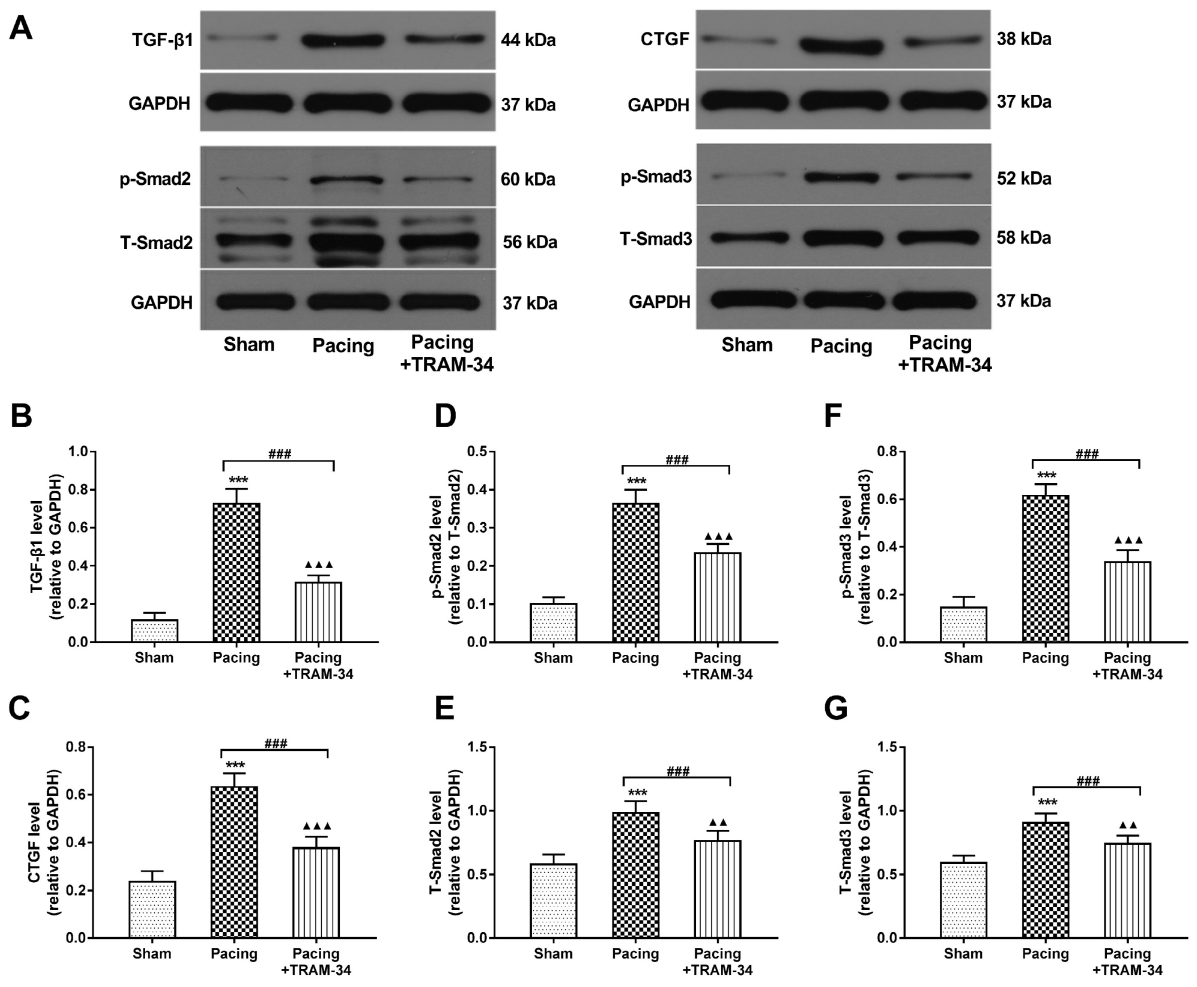
** Effects of SK4 on the expression of CTGF, TGF-β1, Smad2, p-Smad2, Smad3 and p-Smad3 in atria**. (A) Representative Western blot images of CTGF, TGF-β1, Smad2, p-Smad2, Smad3 and p-Smad3 expression levels in the atria. (B-G) The mean levels of CTGF, TGF-β1, Smad2, p-Smad2, Smad3 and p-Smad3 in the atria. ^***^P<0.001 versus the sham group. ^###^P<0.001 versus the pacing group. ^▲▲^P<0.01 versus the sham group. ^▲▲▲^P<0.001 versus the sham group. The data are presented as the mean ± SD, n=6.

ELISA was performed to evaluate the levels of NE and Ang II in plasma and atrial tissue. Compared with the baseline, the levels of NE and Ang II were both markedly increased 7 days after pacing in the pacing group (both P<0.001). Compared with the sham group, the levels of NE and Ang II in the pacing group were significantly increased 7 days after pacing (both P<0.001) and were reduced by the administration of TRAM-34 (both P<0.001) (**Table 1**). The results illustrated that SK4 blockade reduced systemic and local catecholamine and Ang II levels after rapid atrial pacing. Western blot analysis was performed to evaluate the levels of CTGF and TGF-β1 in atria. As shown in **Figure 7A-C**, the levels of CTGF and TGF-β1, important inflammatory mediators that induce fibrosis, were significantly increased in the pacing group compared with the sham group (both P<0.001). Furthermore, the levels of Smad2, Smad3 and their phosphorylated forms, the downstream signaling molecules of TGF-β1, were also markedly elevated in atria in the pacing group compared with the sham group (both P<0.001). All these changes were reversed by TRAM-34 treatment (all P<0.001) (**Figure 7D-G**).

### Blockade of SK4 channels suppressed the MAPK (p38 and ERK1/2) signaling pathway, thereby inhibiting cardiac fibroblast activation and atrial fibrosis

**Figure 8:**
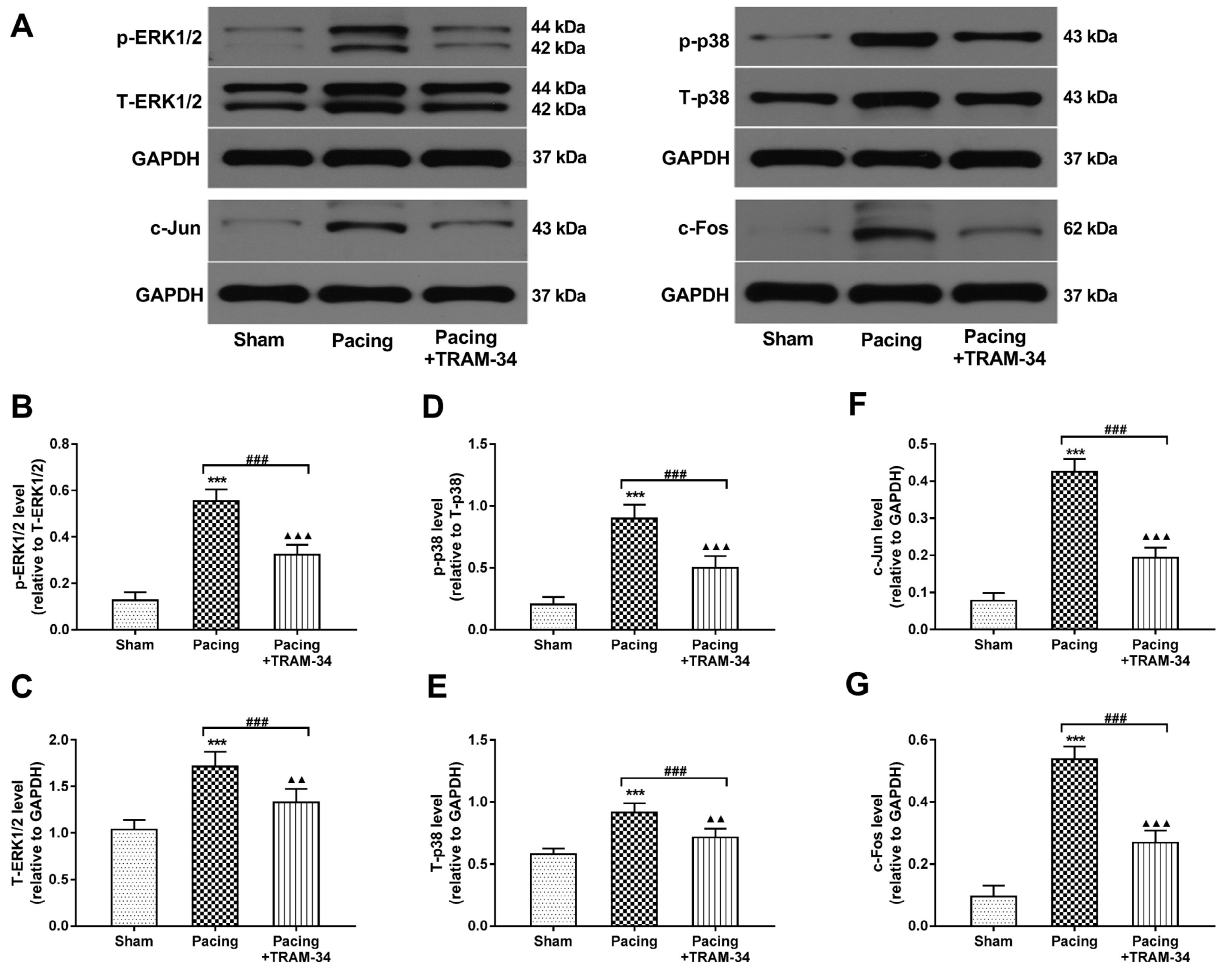
** Effects of SK4 on the expression of ERK1/2, p-ERK1/2, p38, p-p38, c-Fos and c-Jun in atria.** (A) Representative Western blot images of ERK1/2, p-ERK1/2, p38, p-p38, c-Fos and c-Jun expression levels in atria. (B-G) The mean levels of ERK1/2, p-ERK1/2, p38, p-p38, **c-Fos and c-Jun** in atria. ^***^P<0.001 versus the sham group. ^###^P<0.001 versus the pacing group. ^▲▲^P<0.01 versus the sham group. ^▲▲▲^P<0.001 versus the sham group. The data are presented as the mean ± SD, n=6.

**Figure 9:**
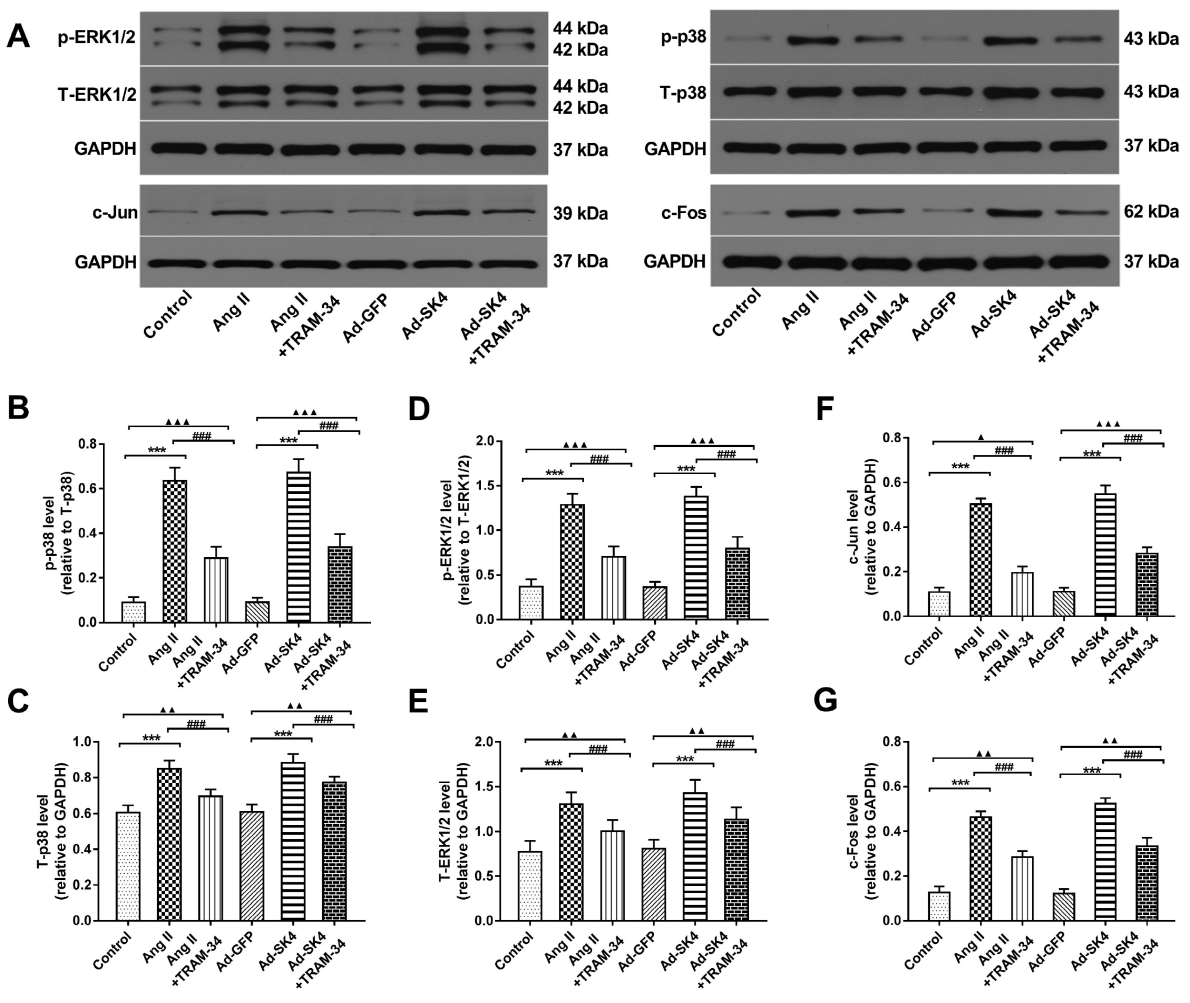
** Effects of SK4 channels on the expression of ERK1/2, p-ERK1/2, p38, p-p38, c-Fos and c-Jun in atrial fibroblasts.** (A) Representative Western blotting images of ERK1/2, p-ERK1/2, p38, p-p38, c-Fos and c-Jun expression levels in cardiac fibroblasts. (B-G) The mean levels of ERK1/2, p-ERK1/2, p38, p-p38, c-Fos and c-Jun in cardiac fibroblasts. ***P<0.001; ^###^P<0.001; ^▲^P<0.05; ^▲▲^P<0.01; ^▲▲▲^P<0.001. The data are presented as the mean ± SD, n=6.

Compared with the sham group, the levels of p38, ERK1/2 and their phosphorylated forms were markedly increased in atria in the pacing group (P<0.001) and were significantly reduced by the administration of TRAM-34 (P<0.001) (**Figure 8A-E**). In addition, the expressions of the downstream transcription factors c-Fos and c-Jun were also significantly elevated in atria of the pacing group compared with the sham group (P<0.001) and were suppressed by TRAM-34 treatment (P<0.001) (**Figure 8A, 8F** and **8G**). To illustrate that the downregulation of p38 and ERK1/2 by TRAM-34 treatment was not entirely related to a decrease in fibrotic factors such as Ang II, we also examined the levels of these proteins *in vitro*. The results showed that the expressions of p38, ERK1/2 and their phosphorylated forms in cardiac fibroblasts treated with Ang II stimulation and Ad-SK4 transfection were significantly increased than those in the control group and the Ad-GFP group (P<0.001), which were dramatically suppressed by TRAM-34 treatment (P<0.001) (**Figure 9A-E**). The changes in the levels of transcription factors c-Fos and c-Jun were identical to the former (Figure **9A**, **9F** and **9G**).

## Discussion

In our previous study, we demonstrated that the expression of SK4 in atria increased significantly in canines after rapid atrial pacing and that SK4 blockade completely inhibited AF induction after 7 h of rapid atrial pacing. In this study, we further explored the role of SK4 in AF maintenance in a canine model of prolonged atrial pacing and provided evidence of the following: (1) the expression of SK4 in atria was significantly increased, and SK4 blockade significantly reduced the AF durations after prolonged rapid atrial pacing; (2) SK4 blockade significantly reduced systemic and local levels of NE and Ang II; and (3) Upregulation of SK4 channels played an important role in the activation of atrial fibroblasts, while SK4 blockade significantly reduced the levels of fibrotic mediators in atria and might affect the function of atrial fibroblasts by blocking p38-MAPK and ERK1/2-MAPK signaling pathway, thereby alleviating atrial fibrosis caused by AF.

SK4, a KCa family member, plays a vital role in signaling cascades during the proliferation, activation, cytokine secretion, and volume regulation of immune cells 18,19. In recent years, SK4 distribution in the heart and its role in automaticity and arrhythmias have been increasingly reported 8-10,20,21. However, in contrast to small conductance KCa with AF, the association between SK4 and AF has rarely been investigated. In our previous studies, we found that the expression of SK4 in atria significantly increased after 7 h of atrial rapid pacing and that SK4 inhibition by TRAM-34 significantly reduced AF vulnerability 11. In the present study, we also discovered that high expression of SK4 in the atria was related to a shortened AERP and an increased dAERP, which were reversed by TRAM-34 treatment. These results suggested that the high expression of SK4 in these atria might have been involved in the mechanism that triggers AF. Atrial conduction abnormity plays an important role in the increased AF stability. In the present study, we found the intra-atrial CT was increased after the atrial rapid pacing and was shortened by TRAM-34 treatment. However, we did not measure conduction velocities in different area, which may be a more convincing explanation for the formation of intra-atrial reentry.

In this study, we focused on exploring the role of SK4 in atrial structural remodeling (atrial fibrosis) induced by AF. Atrial fibrosis is pivotal to the re-entry drive that leads to the maintenance of AF. The content and distribution of fibrous tissue in atria are related not only to the mechanism of AF but also positively to the risk of various complications and treatment failure in patients 22,23. Therefore, the strategy of using atrial fibrosis as a target may provide an important idea for AF treatment. The process of atrial fibrosis is complex and mainly manifests as the imbalanced production of ECM proteins through a variety of signaling systems, such as collagen, the collagen precursor procollagen and collagen processing enzymes 24. In our present study, we found that SK4 blockade significantly reduced the collagen content and MMP levels in atria, thereby suppressing the persistence of AF in canines.

The activation and proliferation of cardiac fibroblasts play important roles in the atrial fibrotic process. Fibroblasts are small, spindle-shaped cells that control the composition and structure of the ECM. Under fibrotic stimulation, fibroblasts proliferate and differentiate into secretory myofibroblasts, which express high levels for the markers α-SMA and DDR-2 16,25. In the present study, we used immunofluorescence staining to detect the expression of SK4 channels in atrial fibroblasts and the number of myofibroblasts in atria, and found that the upregulation of SK4 channels in atrial fibroblasts was associated with the increased number of atrial myofibroblasts, while SK4 blockade dramatically reduced the number of myofibroblasts in atria after prolonged rapid atrial pacing. These results indicated that SK4 played a pivotal role in the activation and proliferation of atrial fibroblasts during AF.

Fibrogenic signaling involves multiple complex molecular signaling systems that interact with each other. *In vitro* and *in vivo* experiments, Ang II, which plays a key role in the RAAS, has been proven to be the central mediator for inducing cardiac fibrosis in AF 26,27. The increase in Ang II expression is closely related to excessive sympathetic activity during AF. In addition, Ang II prompted the synthesis of two other important mediators that cause fibrosis, TGF-β1 and CTGF, by mediating the upregulation of SK4 in cardiac fibroblasts. The relationship between them is interactive and combines positive feedback 14. Interestingly, in our previous study, we found that the reduced expression of SK4 in atria was related to the decreased sympathetic activity 11. In the present study, we found that TRAM-34 treatment suppressed the systemic and local levels of NE and Ang II, which suggested SK4 blockade possibly played a similar role to sympathetic nerve ablation, reducing sympathetic activity during AF. In addition, the increased levels of TGF-β1 and CTGF in atria induced by rapid pacing were also suppressed by TRAM-34 treatment. The downstream signaling of these fibrogenic mediators involves many common intermediates. For example, Ang II promotes fibrotic MAPK phosphorylation (p-p38, p-ERK1/2, etc.) through Ang II receptors, thus activating transcription factors (c-Jun, c-Fos, etc.), which can also be activated by the CTGF signaling system and Smad2/3 pathway induced by TGF-β1 28. In the present study, we found that TRAM-34 treatment significantly attenuated the phosphorylation of p38, ERK1/2 and Smad2/3 in atria after rapid atrial pacing in canines.

To demonstrate that these above effects are not completely associated with the decreased level of Ang II, we further explored the effects of SK4 channels on the function of atrial fibroblasts *in vitro*. We found that atrial fibroblasts treated with Ang II stimulation or Ad-SK4 transfection had the increased expression and current density of SK4 channels, thereby promoting the high expression of α-SMA. These changes could be reversed by SK4 blockade. In addition, after upregulating SK4 channels in atrial fibroblasts through Ad-SK4 transfection, the phosphorylation of p38 and ERK1/2 proteins, along with their transcription factors c-Fos and c-Jun increased, basically consistent with the results in fibroblasts treated with Ang II stimulation. However, SK4 blockade significantly inhibited the expressions of these proteins. The above results indicated that SK4 channels played a critical role in the activation of atrial fibroblasts, which might be achieved by enhanced the p38-MAPK and ERK1/2-MAPK signaling pathways.

**Figure 10:**
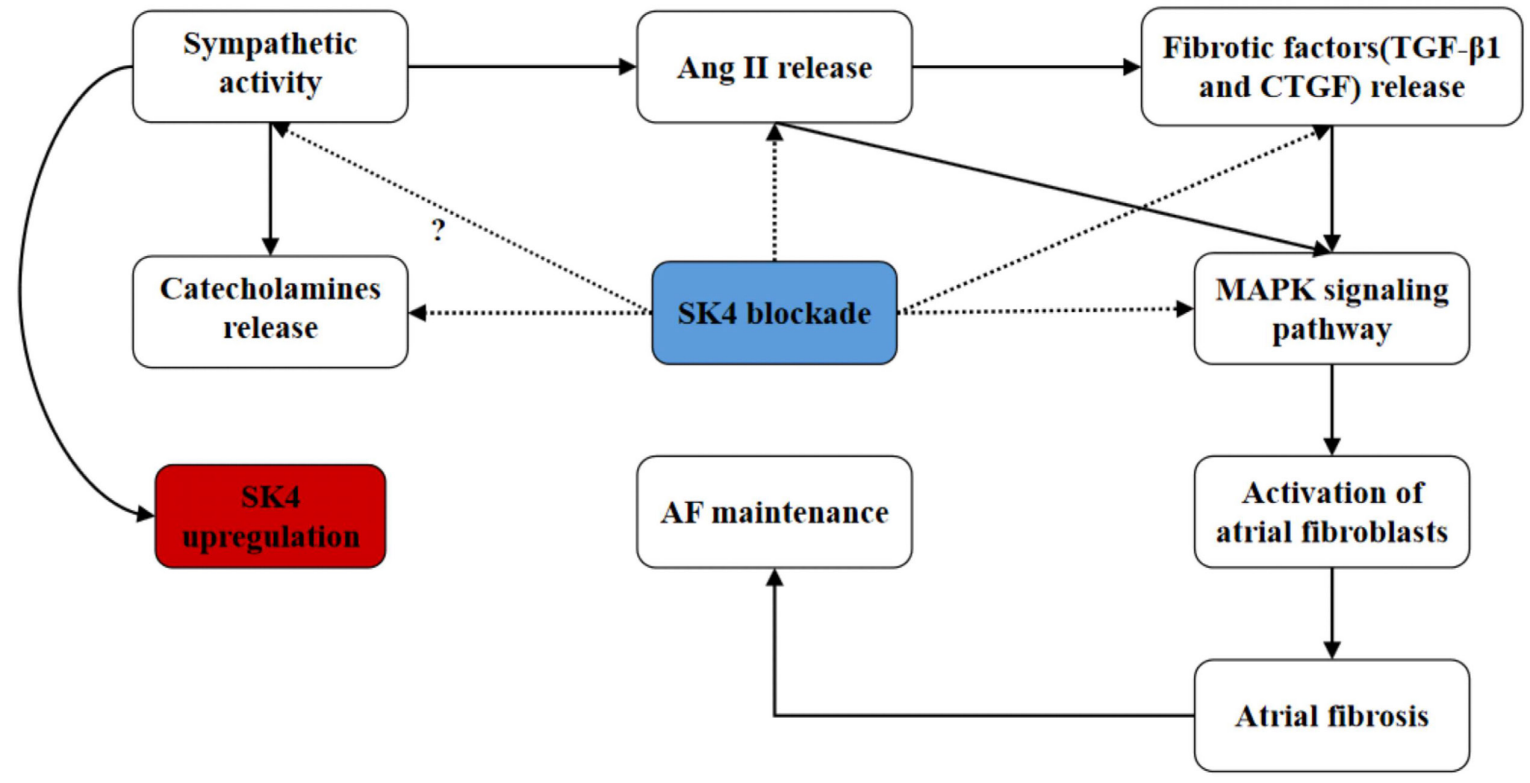
** Schematic diagram: the potential roles of SK4 channels in AF maintenance.** Dotted arrow: negative effect. Solid arrow: positive effect

In summary, we found that the role of SK4 channels in atrial fibrosis during AF might involve multiple pathways (**Figure 10**). On the one hand, SK4 inhibition reduced the catecholamine levels and the release of fibrotic mediators such as Ang II, TGF-β1 and CTGF. On the other hand, SK4 channel also played a role in enhancing the signaling pathway mediating the atrial fibrosis. SK4 Inhibition could effectively attenuate the p38-MAPK and ERK1/2-MAPK signaling pathway, thus inhibiting the activation of atrial fibroblasts and preventing the maintenance and development of AF.

## Clinical prospects

According to the available studies, SK4 channels may play important roles in both the triggering and maintenance of AF, which further elucidating the mechanism of AF. It is expected to become a predictor and a potential therapeutic target for AF, providing the possibility to take SK4 as a novel intervention target for the clinical treatment of AF. However, the effectiveness and safety of SK4 blockade in AF treatment needs further verified. In addition, specific drugs for SK4 blockade such as TRAM-34 and Senicapoc are currently expensive. So, the economic applicability in practical use also needs considered.

## Study limitations

First, our previous study showed that the upregulation of SK4 in atria was closely associated with sympathetic activity. In this study, we observed that the systemic administration of the SK4 inhibitor TRAM-34 significantly suppressed the systemic and local catecholamine levels. However we failed to detect the sympathetic activity. We speculated that the SK4 blockade played a similar role to the sympathetic ablation. Second, in this study we did not investigate the changes in Ca^2+^ levels, nor did we investigate the effects of SK4 activation on atrial myocyte currents after rapid atrial pacing.

## Funding

This work was supported by the National Natural Science Foundation of China (grant number 81670303 and 81970277).

## Figures and Tables

**Table 1:** Levels of NE and Ang II in plasma and atria.

Groups		NE in plasma	NE in atrium	Ang II in plasma	Ang II in atrium
**Sham group (n=6)**	Baseline	268.5±32.6		105.7±11.3	
	7-day time control	279.7±34.2	166.6±32.8	113±13.5	269.2±30.9
**Pacing group (n=7)**	Baseline	265.7±33.9		108.2±11.4	
	7-day pacing	562.4±51.4^***^###	729.2±55.1^***^	250.7±16.8^***^###	722.4±45.7^***^
**Pacing+TRAM-34 group (n=7)**	Baseline	270.3±36.2		109.4±10.5	
	7-day pacing	386.9±38.6&&&▲▲▲	309.7±41.7&&&▲▲▲	165.6±16.5&&&▲▲▲	372.1±40.1&&&▲▲▲

^***^P<0.001 versus the baseline; ###P<0.001 versus the same time in the sham group; &&&P<0.001 versus the same time in the pacing group; ▲▲▲P<0.001 versus the same time in the sham group. **Abbreviations:** NE, norepinephrine; Ang II, angiotensin II.
